# Antioxidant and Antimicrobial Activities of Crude Extracts and Fractions of Cashew (*Anacardium occidentale* L.), Cajui *(Anacardium microcarpum)*, and Pequi (*Caryocar brasiliense* C.): A Systematic Review

**DOI:** 10.1155/2018/3753562

**Published:** 2018-04-18

**Authors:** Anderson Baptista, Reggiani Vilela Gonçalves, Josefina Bressan, Maria do Carmo Gouveia Pelúzio

**Affiliations:** ^1^Department of Nutrition and Health, Universidade Federal de Viçosa, 36570-900 Viçosa, MG, Brazil; ^2^Department of Animal Biology, Universidade Federal de Viçosa, 36570-900 Viçosa, MG, Brazil

## Abstract

The accentuated increase in the use of medicinal plants by the population to treat diseases makes it necessary to carry out pharmacological studies in order to contribute to the scientific knowledge and clarify the mechanisms involved in the main compounds present in these plants. Due to the difficulty of combating antimicrobial-resistant microorganisms, plants become a low-cost and effective alternative. The stem, fruit, and leaves of plants are used to measure antioxidant and antimicrobial capacity and to combat the oxidative degradation of free radicals produced in the presence of xenobiotics. A systematic review is a powerful tool that incorporates the variability among the studies, providing an overall estimate of the use of plant extracts as antioxidants and antimicrobial activities. In view of the controversies in the literature regarding the use of compounds from plants or the isolation and purification of the main substances for the prevention of bacterial various therapeutic actions, the aim of this was to present a systematic review on the antimicrobial and antioxidant properties of cashew *(Anacardium occidentale)*, cajui *(Anacardium microcarpum)*, and pequi *(Caryocar brasiliense)*. The following databases were analyzed: PubMed/Medline, Virtual Health Library (LILACS and SciELO), and Science Direct. Out of 425 articles, 33 articles have been used in this study, which were also represented in the Prisma Statement. *In vitro* antioxidant tests were conducted in 28 studies using different methodologies. Most of the tests involving the studied species demonstrated positive antioxidant potential and antimicrobial properties. The results provide important data and perspectives into the use of natural products that can contribute to the treatment of various diseases.

## 1. Introduction

Plants have long been used for the prevention and treatment of human health adversities. The first herbal records date back to 2838–2698 B.C., when the Chinese emperor Shen Nung cataloged 365 medicinal herbs. In 1500 B.C., the Egyptian manuscript “Ebers Papyrus” recorded information on 811 prescriptions and 700 drugs. Some of these plants are still in use, such as ginseng *(Panax* spp.), *Ephedra* spp., *Cassia* spp., and *Rheum palmatum* L., being used as a source of drugs for the pharmaceutical industry. Indigenous tribes in their rituals and cure of diseases have always used medicinal plants [1].

The use of phytotherapy started gaining popularity in the mid-70s and 80s. The trade of herbal medicines in Brazil is around 5% of the total trade of medicines [2]. According to the Ministry of Health, patients seeking treatment based on medicinal plants and phytopharmaceuticals increased to 161% between 2013 and 2015, probably due to the low cost of herbal medicines and also to the fact of the population being accustomed to their use [3]. The World Health Organization (WHO) notes that 70% to 95% of the population depend on the use of herbal medicines in the primary care setting, therefore issuing a recommendation to encourage countries to formulate national policies and regulations regarding the use of traditional medicines with proven effectiveness [4].

The concept of medicinal plants being “natural” does not guarantee benefits and safety, which makes it fundamental that a popularly known herbal medicine is widely studied with regard to its pharmacological and toxicological aspects in order to understand its adverse effects [5]. Adverse effects arise from the production of plant secondary metabolites that can be toxic to the organism, as anthraquinone, for instance, in *Aloe vera* can cause nephritis when the latter is ingested in a high concentration. In addition, the pyrrolizidine alkaloid metabolites present in comfrey *(Symphytum officinale)* are also hepatotoxic [6]. The appearance and dissemination of microorganisms resistant to commercially available antimicrobials have been reported for decades, encouraging the search for new sources of antimicrobial substances, such as plants used in the traditional medicine and laboratory trials [7]. The use of plants as antimicrobial agents has seen a major increase in the last years. A good example of this fact is phenolic compounds, present in the essential oils of many plants that are known as active substances, such as the essential oil of rosemary leaves, used in the preservation of food to inhibit microbial contamination and dissemination [8]. Another example is that barks of the cashew tree have shown a considerable bactericidal effect due to the presence of tannins [9].

Apart from antimicrobial agents, the pursuit for safe natural antioxidants that can be beneficial to the human health and can replace those of the synthetic origin is of interest to the scientific community [10]. The plant kingdom is a valuable source of bioactive and phytochemical compounds. Furthermore, the adequate consumption of fruits and vegetables is directly related to the reduced risks of diseases due to the amount of health-beneficial antioxidants present in such plants [11].

The oxidative stress, which occurs in cells, in general, can be combated by antioxidants since they hold oxidation stability and therefore prevent the formation of reactive species of oxygen and nitrogen. Reactive oxygen species such as superoxide radicals, hydroxyl radicals, and hydrogen peroxide may favor the development of diseases such as cancer, cardiovascular disorders, aging, and degenerative diseases. In contrast, the consumption of natural antioxidants, such as polyphenol-rich foods, fresh fruits, and vegetables, can counteract the oxidative degradation of free radicals [12, 13]. In this context, we can highlight 3 plants (caju, cajui, and pequi) which are widely used in cooking and in traditional Brazilian medicine, mainly in the north, northeast, and central west regions of the country. Cashew nut and its byproducts have several industrial and biological properties such as antioxidant and antimicrobial activities. There are 11 different species in the genus *Anacardium*, in which the *Anacardium occidentale* L. (cashew) is the most common in Brazil, especially in the north and northeast regions. This pseudofruit is juicy and rich in vitamin C (200 mg/100 g of juice) [14]. *Anacardium microcarpum* (cajui) is widely used in traditional folk medicine for the treatment of inflammation, rheumatism, tumors, and infectious diseases. The extracts can hold potential antioxidant agents that modify the oxidation states of cells [15]. *Caryocar brasiliense* C. (pequi) is a native plant of the Cerrado biome, and it is well distributed in the north and midwest regions of the country. The fruit has carotenoids with an antioxidant activity and is a precursor of vitamin A [16]. It demonstrates a strong potential for sustainable exploration, since the fruit is fairly rich in a nutritional and functional point of view, presenting sensory properties such as color, aroma, and a distinctive flavor compared to other fruits, besides having a pleasant taste [17].

Some clinical and preclinical studies have attempted to demonstrate the antioxidant and antimicrobial effect of plant compounds and their derivatives. However, this hypothesis may not always be confirmed mainly due to the comprehensive methodological variations involving the obtaining of the compounds, the therapeutic schemes, and the mechanisms of action. However, it is important to search for new data from various studies in order to clarify the aforementioned discrepancies. In this context, the systematic review is a powerful tool that incorporates the variability among the studies and allows obtaining of an overall estimate of the use of plant extracts (cashew, cajui, and pequi) with antioxidant and antimicrobial properties. Moreover, a systematic review, unlike the widely used narrative reviews, has never been carried out before and might provide us with reliable and solid new evidence on whether or not crude extracts and fractions of cashew, cajui, and pequi could be beneficial in antioxidant and antimicrobial defense mechanisms. Based on the latter, our systematic review has been developed to present the results of tests with extracts of parts of the following plant species: *Anacardium occidentale* L., *Anacardium microcarpum*, and *Caryocar brasiliense* C. The hypothesis is that these species contain substances that are beneficial to the human health and could be appropriately used by the population, replacing synthetic products and expanding the National Policy on Integrative and Complementary Practices in Health (PNPIC) of the Brazilian Unified Health System (SUS). The results can then lead to a greater discussion and provide interest to the pharmaceutical industry in reducing the high costs of producing and purchasing synthetic substances [18].

## 2. Methodology

### 2.1. Literature Research

The studies included in this review have been selected using the following databases: PubMed/Medline, Virtual Health Library (BIREME, LILACS, and SciELO), and Science Direct. The descriptors used were “pequi,” “pequi antioxidant,” “antimicrobial pequi,” “*Caryocar brasiliense*,” “Caryocar,” “caju antioxidant,” “cajui antioxidant,” “bacteria caju,” “caju antimicrobial,” “cashew,” “*Anacardium occidentale*,” “cajui,” and “*Anacardium microcarpum*.” The original studies used in this review covered the period from 2006 to 2016. This time period can be justified by the limited number of specific studies conducted in recent years and their relevance. Classic articles on the topic and the others resulting from a reverse search were also selected. Only articles published in English, Portuguese, and Spanish have been included. However, studies that focused on toxicity, wound healing, anti-inflammation, chemical characterization, prebiotic, genotoxic, antidiabetic, gastroprotective, and cardiovascular diseases have been eliminated. Reviews, comments, and notes as well as unpublished studies have not been considered. The studies have been selected based on the inclusion criteria indicated below:

- Studies reporting the effect of antioxidant and antimicrobial of crude extracts, fractions, and metabolite isolated of the cashew tree *(Anacardium occidentale* L.), cajui *(Anacardium microcarpum)*, and pequi *(Caryocar brasiliense* C.) in the animal model
- Studies *in vitro*, reporting the effect of antioxidant and antimicrobial of crude extracts, fractions, and metabolite isolated of the cashew tree *(Anacardium occidentale* L.), cajui *(Anacardium microcarpum)*, and pequi *(Caryocar brasiliense* C.)

### 2.2. Extraction and Data Management

For abstract selection, three independent reviewers (BAB, BJ, and PMC) have selected studies based on the title and abstract analysis. In case of disagreement, a fourth reviewer (GRV) would decide whether the study met the inclusion and exclusion criteria. In order to eliminate subjectivity in the data collection and selection process, the information has been independently extracted by both reviewers (BAB and PMC) and analyzed separately. Data from each study has been extracted and tabulated using standardized information, such as features of the publication (author, country, and year), plant (plant family, species, and popular name and part used), test conducted, type of analysis, test dosage, animal model, number of animals, sex, and type of extract used. When the reviewers faced some kind of difficulty in extracting the data or in obtaining the studies, the authors would be contacted by e-mail to provide the necessary information. Subsequently, the data has been compared, and the conflicting information was identified and corrected through discussion in order to reach consensus among the reviewers.

## 3. Results and Discussion

**Figure 1:**
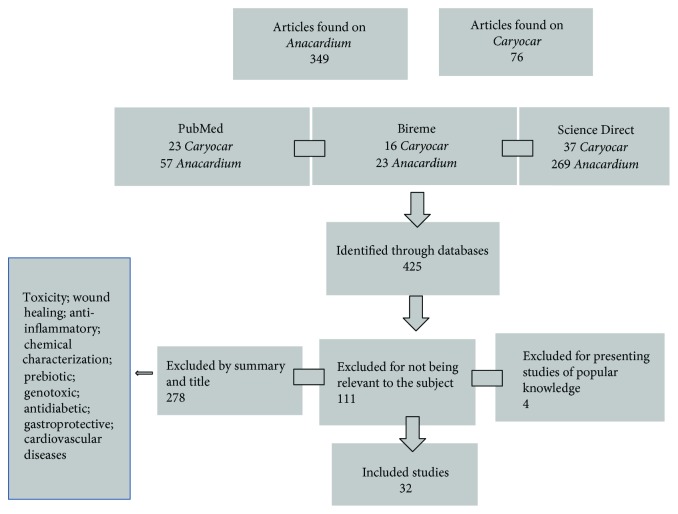
The flow diagram report of the systematic review literature search results.

The initial search generated 425 studies out of which 325 were assigned to the descriptor cashew, 24 for cajui, and 76 for pequi. Studies that have not met the previously defined criteria were disregarded. The articles that did not report antioxidant and/or antimicrobial activity, those related only to popular knowledge, without relevance and literature reviews, were of 392. A total of 32 articles were included at the end of the analysis; other 24 studies performed tests for antioxidant action, 13 ran tests for antimicrobial action, and 5 articles conducted both tests. The Brazilian states that carried out the studies were Ceará (10 studies), Minas Gerais (8 studies), Goiás (1 study), the Federal District (3 studies), Paraíba (2 studies), Mato Grosso (1 study), and Piauí (1 study). Some other studies have also been found in Mexico (1 study), the United States (1 study), Malaysia (1 study), Cuba (1 study), and Africa (2 studies). The exclusion of articles can be justified because they investigate different lines of research from the scope of this study (study flow diagram, shown in Figure 1).

Considering the results shown above, it can be observed that although some countries report the therapeutic use of these extracts, it is in Brazil that most of the works are specific, reporting the beneficial effects of these 3 species to the human health. Possibly this fact can be justified by the regular use of these plants in traditional Brazilian cooking. From this, there are reports in the population of a possible therapeutic power of these extracts, acting mainly through antioxidant, antimicrobial, and regenerative properties. Currently, one of these plants *(A. occidentale)* is already listed in the National Program of Medicinal Plants and Herbal Medicine of the country's unique health system for therapeutic purposes. Considering the similar characteristics of the three extracts, we believe that it will be a matter of time for the other two species *(A. microcarpum* and *C. brasiliense)* to be also added to this list. Furthermore, these 3 plant species have a number of total phenolic compounds as flavonoids, anthocyanins, and tannins [15, 19, 20], which are therapeutically recognized in the treatment of several conditions, such as cancer, cardiovascular diseases, aging, and neurodegenerative illnesses. Epidemiological studies have suggested that the consumption of natural antioxidants such as vitamins, flavonoids, anthocyanins, and other phenolic compounds has protective effects against the previously mentioned diseases [13, 21, 22]. The interventions with herbal and phytotherapeutic plants take place in the primary health care setting. The practice of phytotherapy involves the interaction between knowledge, multiprofessional efforts in health care, prevention, and health actions (Table 1). The results of our work suggest a growing interest for natural products of plant origin in recent years, mainly due to the use of these compounds in health care and prevention (Tables 1 and 2). Several studies have reported relevant results mainly in combating oxidative stress and antimicrobial action. These results highlight the importance and relevance of popular knowledge in the treatment of human diseases using phytotherapies. In 2009, the Ministry of Health made an available list of 71 medicinal plants, which comprise the National Register of Medicinal Plants of Interest to the Unified Health System (RENISUS), being its purpose to boost the generation of products for use mainly in the basic health care setting through the development of the entire productive chain related to the regulation, cultivation, management, production, marketing, and distribution of medicinal plants and herbal remedies.

**Figure 2:**
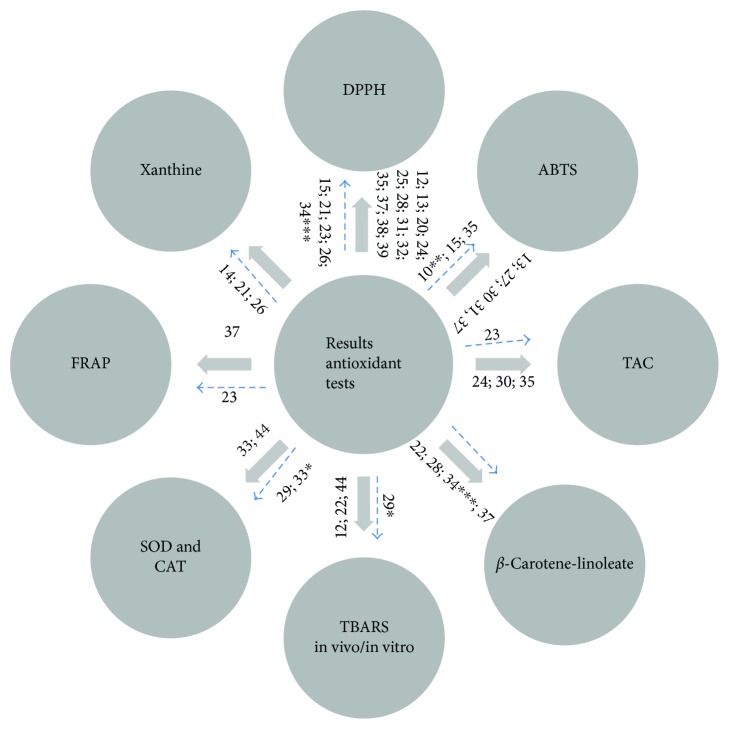
Antioxidant tests used from extract, fractions, oils, and supercritical carbon dioxide (^∗^Anacardic acids; ^∗∗^Supercritical CO_2_; ^∗∗∗^Oil; solid arrows = crude; dashed arrows = fractions).

Our results showed that 27 studies conducted *in vitro* antioxidant tests using different methodologies, the DPPH (2-diphenyl-1-picrylhydrazyl) being the most common followed by ABTS (2,2′-azino-bis-3-ethylbenzothiazoline-6-sulfonic acid) (Figure 2).

The supercritical CO_2_ extraction system consists of a heated extraction column, CO_2_ and cosolvent pumps, a thermostatic bath, and a pressure gauge, which is a nonpolluting method for extracting plant products. In addition to its low toxicity and environmental impact, supercritical CO_2_ extraction replaces conventional extraction methods using organic solvents that require numerous purification processes to remove chemical contaminants [10]. Assays such as *β*-carotene, FRAP, and xanthine have been poorly used probably because they result in difficult numbers to compare, since there is no universal method capable of accurately measuring the antioxidant capacity of all samples.

The determination of the minimum inhibitory concentration in microplate wells was the method most frequently adopted. The antimicrobial test most used was the minimal inhibitory concentration followed by the agar diffusion test and antiseptic test (Table 3). Our results also showed that among the markers of oxidative stress, the most frequent analysis was of thiobarbituric acid markers (TBARS), followed by oxygen radical absorbance capacity (ORAC), total antioxidant capacity (TAC), xanthine oxidase, and analyses of antioxidant enzymes superoxide dismutase (SOD) and catalase (CAT). Basically, the results showed that cashew, cajui, and pequi extracts decreased the production of TBARS in tissues by increasing the total antioxidant capacity and accelerating the formation of hydrogen peroxide (H_2_O_2_) from molecular oxygen (O_2_^−^) by SOD action and also by accelerating the decomposition of H_2_O_2_ by CAT forming water.

Most of the analyzed studies performed *in vitro* activities to demonstrate the antioxidant and antimicrobial potentials of cashew, cajui, and pequi extracts. The tested doses varied substantially, which calls the obtained results into question. Apart from that, there seems to be a lack of information to explain the potential benefits of these extracts to the human health [45]. Another issue that should also be taken into consideration is the significant variation in the reported results using different parts of the plants such as fruits, oils, leaves, and barks. The tests for cashew, cajui, and pequi showed that all parts of the plants offer a therapeutic potential when it comes to antioxidant and antimicrobial activities, pointing out the possibilities for developing therapeutic products of plant origin, thus stimulating new research and increasingly consolidating the use of plants that display therapeutic features.

Our study demonstrated that 12 articles have performed tests to assess the antimicrobial effect of different parts of the plants. According to da Silva et al. [40], the hydroalcoholic extract of the cashew tree bark, in varied doses, was effective in avoiding the proliferation of *Staphylococcus aureus*. Studies have shown that even in small doses, the tannins present in the cashew tree bark are effective in inhibiting the proliferation of this bacterium [9, 19]. The effects of these extracts on other bacteria such as *Pseudomonas aeruginosa*, *Escherichia coli*, and *Streptococcus* spp. have also been analyzed, and the results showed that cajui and pequi extracts inhibited the proliferation of such bacteria [38]. This activity was related to the high concentration of flavonoids, tannins, and alkaloids present in the extracts [42]. Similarly, the variations in the results can be justified by the different concentrations of these compounds in different parts of the plants, like leaves, barks, and essential oils [34]. This growing need to discover new natural antibiotics simultaneously arises from the ever increasing resistance of these bacteria to the most common antimicrobials, such as penicillin. Therefore, the development of alternative plant-based drugs is urgent and essential in the fight against microbial agents [46].

In our study, 16 articles showed the antioxidant action of the extracts after analyzing leaves, fruits, fibers, and oils obtained from cashew, cajui, and pequi. For dos Santos et al. [21], the cashew extract serves as an electron donor, acting as a primary antioxidant that accelerates the passage of electrons, quickly stabilizing molecules. The cashew peduncle extract was used to evaluate the formation of TBARS in the liver, plasma, and brain to determine the lipid peroxidation level in the tissue. The results showed an 80% decrease in the formation of malondialdehyde and a 95% increase in total antioxidant capacity. Interestingly enough, cashew peduncles are usually disposed of and, due to that, it is one of the least valued parts of the fruit. Perhaps, it could represent a low-cost alternative in the production of new medicines in the future [12]. Another antioxidant function attributed to the cashew extract is the increase in the activity of SOD and CAT antioxidant enzymes and, consequently, a decrease in lipid peroxidation, reducing damages to cell membranes [29].

However, it is clear that the results vary according to the part of the plant studied. For instance, when using the DPPH technique for radical elimination activity, it has been observed that cashew fruits have a high antioxidant power, which can vary according to the place they were cultivated [28]. Anacardic acids and cardanol extracted from the cashew oil did not inhibit lipid peroxidation, probably because they do not possess the ability to donate the hydrogen atom to the peroxy radical, derived from the free fatty acid. Nevertheless, the anacardic acid inhibited the formation of superoxide anions and the ability of various enzymes involved in promoting free radicals in the tissue [26]. The antioxidant power of the leaves (TAC) was measured through the FRAP technique (phosphomolybdenum and ferric reducing antioxidant power techniques). The microbicide test with bacteria and fungi showed little effectiveness against bacteria activity and no effectiveness against fungi, with better results for gram-negative bacteria [23]. Cashew nut bran was also evaluated in different stages, raw and cooked, by Soares et al. [31], using DPPH and ABTS. When comparing the different stages of bran, they observed that the raw kind presents the greatest antioxidant activity. Cashew fruit and nuts have been evaluated by the hypoxanthine/xanthine oxidase test, and they demonstrated high antioxidant capacity with 100% inhibition obtained by the liquid extract of the nut and 94% inhibition by the fiber. The anacardic acids had the highest antioxidant activity when compared to cardol and cardonol. Anacardic acids present in large quantities in the cashew fibers (residue of cashew nut extraction) can be utilized for the production of chemopreventive substances and protectors of DNA damage instead of being disposed of [14]. Breda et al. [43] have observed that extracts of the fruit peels and leaves of pequi displayed antifungal activity against several species of fungi, with better efficiency observed in the peel extracts. This difference can be justified by the presence of phenolic phytochemicals in a greater amount in the fruit peels.

In the case of pequi, Morais et al. [37] have observed that the mesocarp acts as a radical collector, providing a reduction of Fe^+3^ when compared to other plants such as *Cipocereus minensis*, *Solanum lipocarpo*, and *Byrsonina verbascifolia*. The antioxidant activity of the pequi leaf is comparable to the ones found in isolated compounds of rutin and vitamin C [38]. For de Pinho et al. [44], the pequi oil stimulated the antioxidant defense system, increasing the activity of antioxidant enzymes SOD, CAT, and glutathione peroxidase (GPX) after the induction of lipid peroxidation by CCl4 application. According to Khouri et al. [35], the fruit extract reduced hydroxyl radicals, inhibiting Fenton's reagent, an important way to form free radicals in tissues. Our results show that any part of the plant used has a high antioxidant power, by acting positively in all ways of forming free radicals and stimulating antioxidant defense systems. Breda et al. [43] have observed that extracts of the fruit peels and leaves of pequi presented antifungal activity against several species of fungi, with better efficiency observed in the peel extracts. This difference can be justified by the presence of phenolic phytochemicals in a greater amount in fruit peels.

## 4. Limitations

Although our systematic review represents a proposal to compile and critically analyze the evidence on the applicability of plant derivatives (cashew, cajui, and pequi) as an antioxidant and antimicrobial, a limitation of the results should be considered. Our sampling frame was based on a specific number of databases. Thus, some articles may be not recovered due to the boundaries applied in the search strategy, as well as limitations in algorithms adopted in the search interfaces of each database. These aspects directly affect the sensitivity and specificity of the search strategy, which may have contributed to identify key articles. We attempted to reduce these limitations by screening the reference lists of all articles, which are not limited to databases or any keyword-based search model. In addition, most of the studies were identified to be conducted in the same country, Brazil, which may be related to the failure in searching for studies.

## 5. Conclusion

The parts of pequi and cashew trees can be used to treat infectious diseases caused by bacteria and fungi and to fight free radicals. The DPPH technique was the most utilized, and it demonstrates that, along with other techniques, the extracts show a satisfactory antioxidant power and in vivo actions that provide protection from oxidative processes. The isolated secondary metabolites suggest better antioxidant activity in relation to the crude extract, such as anacardic acids from cashew. The ethyl acetate fraction suggests having the best antioxidant and bactericidal action. The antimicrobial activities of the extracts in bacteria and fungi proved their efficiency, primarily for minimum bactericidal concentration testing. The studies mostly used crude extracts. However, the isolated secondary metabolites may have more potent antioxidant and microbicidal action. Based on this, we believed that researches on actions of cashew, cajui, and pequi are important for the treatment of populations, mainly for reducing costs and increasing the therapeutic spectrum. Furthermore, the use of herbal medicines can also arouse the interest of the industry, adding new value to the pharmaceutical market. However, the absence or incomplete characterization of the models, experimental groups, treatment protocols, phytochemical screening, and toxicity analysis of the plant products impairs the internal validity of the individual studies. Together with these limitations, contradictory results based on heterogeneous studies of the same plant species compromise the external validity of the evidence, making it difficult to translate data into clinical practice, as well as the relevance of the plant species as potential biotechnological targets in the development of new drugs.

## Figures and Tables

**Table 1:** Antioxidant properties and main analysis of studies found citing cashew, cajui, and pequi.

Species, family, and popular name	Parts used	Antioxidant assay	Analysis	Dose of the test	Country	Animal model	Number of groups	Sex	Extract used	References
*Anacardium occidentale* L. Anacardiaceae Cashew	Leaves	FRAP; DPPH; TAC	*In vitro*	1 mg/mL	Nigeria	—	—	—	Fraction	Ajileye et al., 2015 [23]
*Anacardium occidentale* L. Anacardiaceae Cashew	Fruit	DPPH; TAC	*In vitro*	?	Brazil	—	—	—	Crude	Alves et al., 2013 [24]
*Anacardium occidentale* L. Anacardiaceae Cashew	Cashew nut	DPPH; xanthine	*In vitro* *In vivo* antioxidant assay (the *Saccharomyces cerevisiae* model)	100, 200, 500, and 1000 *μ*g/mL	Brazil	—	—	—	Cashew nut shell liquid	[21]
*Anacardium occidentale* L. Anacardiaceae Cashew	Fruit	DPPH; TBARS	*In vitro* *In vivo*	200/400 mg/kg	Brazil	Rats	24	Males	Crude	[12]
*Anacardium occidentale* L. Anacardiaceae Cashew	Fruit	TPC; BETA CAR/LIN; TBARS	*In vitro*	0.5 mg/mL	Sri Lanka	—	—	—	Crude	[22]
*Anacardium occidentale* L. Anacardiaceae Cashew	Stem bark	DPPH; TPC	*In vitro* *In vivo*	40.2, 127, and 402 mg/kg	Africa	Mice	28 mice 7 groups (*n* = 4)	Males	Crude	[25]
*Anacardium occidentale* L. Anacardiaceae Cashew	Stem bark	DPPH; xanthine	*In vitro*	?	United States of America	—	—	—	Fractions	[26]
*Anacardium occidentale* L. Anacardiaceae Cashew	Fibers and fruit	ABTS; TPC	*In vitro*	500 mL juice	Brazil	—	—	—	Crude	[27]
*Anacardium occidentale* L. Anacardiaceae Cashew	Fruit	DPPH; BETA CAR/LIN	*In vitro*	20–300 g pulp fruit 1 : 2 water	Brazil	—	—	—	Crude	[28]
*Anacardium occidentale* L. Anacardiaceae Cashew	Fruit peels	DPPH; TSP; ABTS; AOC	*In vitro*	1 g of freeze-dried peel	Mexico	—	—	—	Crude	Moo-Huchin et al., 2015 [13]
*Anacardium occidentale* L. Anacardiaceae Cashew	Fruit peels	Gastric nitrate/nitrite levels; SOD; CAT; TBARS	*In vivo*	30 mg/kg	Brazil	Mice and rats	8 animals per group	Males	Anacardic acids	[29]
*Anacardium occidentale* L. Anacardiaceae Cashew	Fruit peels	TAC; TPC; ABTS	*In vitro*	?	Brazil	—	—	—	Crude	[30]
*Anacardium occidentale* L. Anacardiaceae Cashew	Fruit	DPPH; ABTS; TPC	*In vitro*	1 g	Brazil	—	—	—	Crude	[31]
*Anacardium occidentale* L. Anacardiaceae Cashew	Leaves	DPPH; TPC; FRP	*In vitro*	0.3 and 1.0 g/50 mL of methanol	Malaysia	—	—	—	Crude	[32]
*Anacardium occidentale* L. Anacardiaceae Cashew	Nut, fiber, and fruit	Xanthine	*In vitro*	10 mg/mL	Brazil	—	—	—	Fractions	[14]
*Anacardium microcarpum* Anacardiaceae Cajui	Stem barks	DPPH; TBARS	*In vitro* *In vivo*	1–400 *μ*g/mL	Brazil	Rats	?	?	Fractions	[15]
*Anacardium microcarpum* Anacardiaceae Cajui	Stem barks	TPC; ABTS; SOD; CAT; GST	*In vitro*	1–400 *μ*g/mL, 1, and 10 mg/mL	Brazil	—	—	—	Crude/fractions	[33]
*Caryocar brasiliense* Caryocaracea Pequi	Leaves	ABTS; human fibroblast culture	*In vitro*	0.2–0.025% *w*/*v*	Brazil	—	—	—	Supercritical CO_2_	[10]
*Caryocar brasiliense* Caryocaracea Pequi	Oil	DPPH; TAC; BETA CAR/LIN	*In vitro*	0.2 g/L	Brazil	—	—	—	Oil	[34]
*Caryocar brasiliense* Caryocaracea Pequi	Oil	DPPH; TPC; ILP; HCA; TAC	*In vitro*	?	Brazil	—	—	—	Crude	[20]
*Caryocar brasiliense* Caryocaracea Pequi	Fruits	TPC; TBARS	*In vivo*	0.1 g/mL	Brazil	Mice	10 gr. with 8 anim	Both	Crude	[35]
*Caryocar brasiliense* Caryocaracea Pequi	Fruits	TBARS	*In vivo*	0.5 mL·kg^−1^ and 1.0 mL·kg^−1^	Brazil	Mice	6 gr. with 8 anim	Both	Crude	[36]
*Caryocar brasiliense* Caryocaracea Pequi	Fruits	DPPH; ABTS; FRAP; BETA CAR/LIN	*In vitro*	0.5, 1.0, and 1.5 mg/mL	Brazil	—	—	—	Crude	[37]
*Caryocar brasiliense* Caryocaracea Pequi	Leaves	DPPH	*In vitro*	10.0 mg/mL	Brazil.	—	—	—	Crude	[38]
*Caryocar brasiliense* Caryocaracea Pequi	Oil	TPC; TBARS; ORAC; SOD; CAT; GPX	*In vivo*	3 mL/kg	Brazil	Rats	40	Males	Crude	[39]

? = not informed; gr = groups; anim = animals; ABTS = 2,2′-azinobis-3-ethylbenzotiazoline-6-sulfonic acid; AOC = antioxidant capacity; BETA CAR/LIN = *β*-carotene-linoleate model system; xanthine = hypoxanthine/xanthine oxidase assay; DPPH = radical scavenging assay; FRAP = ferric reducing antioxidant power; FRP = ferric reducing power; ORAC = oxygen radical absorbance capacity; TAC = total anthocyanin content; TPC = total phenolic content; TSP = total soluble phenols; TBARS = thiobarbituric acid reactive substance; HCA = total hydroxycinnamic acid content; ILP = inhibition of lipid peroxidation; SOD = superoxide dismutase; CAT = catalase; GPX = glutathione reductase; GST = glutathione-S-transferase.

**Table 2:** Antimicrobial properties and main analysis of studies found citing cashew, cajui, and pequi *in vivo* and *in vitro*.

Species, family, and popular name	Parts used	Antimicrobial assay	Analysis	In vivo	Dose of the test	Country	Tested microorganism	Extract used	References
*Anacardium occidentale* L. Anacardiaceae Cashew	Leaves	Agar diffusion test	*In vitro*	—	50–200 mg/mL	Cuba	*Staphylococcus aureus*; *Bacillus subtilis*; *Salmonella entérica*; *Shigella* sp.; *Escherichia coli*	Crude/fractions	[19]
*Anacardium occidentale* L. Anacardiaceae Cashew		Agar diffusion test/^∗^MIC	*In vitro*	—		Nigeria	*Escherichia coli*; *Pseudomonas aeruginosa*; *Staphylococcus aureus*; *Proteus mirabilis*; *Bacillus subtilis*; *Klebsiella pneumoniae*; *Clostridium sporogens*; *Candida albicans*; *Candida pseudotropicalis*	Fraction	Ajileye et al., 2015 [23]
*Anacardium occidentale* L. Anacardiaceae Cashew	Fruit peels	MIC	*In vitro*	—	50 *μ*g/mL	Brazil	*Staphylococcus aureus*	Fraction	[9]
*Anacardium occidentale* L. Anacardiaceae Cashew		MIC	*In vitro*	—	100–0.19 mg/mL	Brazil	*Staphylococcus aureus*	Crude	[40]
*Anacardium occidentale* L. Anacardiaceae Cashew	Stem bark	Agar diffusion test	*In vitro*	—	12.5% and 50%	Brazil	*Streptococcus mitis*; *Streptococcus mutans*; *Streptococcus sanguis*; *Streptococcus sobrinus*	Crude	[41]
*Anacardium occidentale* L. Anacardiaceae Cashew	Leaves		*In vitro*	—	3 g and 10 g/100 mL of methanol	Malaysia	*Brevibacillus brevis*; *Micrococcus luteus*; *Staphylococcus cohnii*; *Escherichia coli*; *Pseudomonas aeruginosa*; *Salmonella enterica*	Crude	[32]
*Anacardium microcarpum* Anacardiaceae Cajui	Stem barks	MIC; modulationof the antibiotic activity	*In vitro*	—	1024 *μ*g/mL	Brazil	*Escherichia coli*; *Pseudomonas aeruginosa*; *Staphylococcus aureus*	Fractions	[42]
*Caryocar brasiliense* Caryocaracea Pequi	Leaves	MIC; antiseptic activity	*In vitro*	—	11.25–100 mg/mL	Brazil	*Escherichia coli*; *Pseudomonas aeruginosa*; *Staphylococcus aureus*	Supercritical CO_2_	[10]
*Caryocar brasiliense* Caryocaracea Pequi	Fruits and leaves	MIC; ^∗∗^MFC	*In vitro* *In vivo*	Acute oral toxicity evaluation of the most active extract; female mice, Swiss at the age of 8 weeks	2000 and 1.95 *μ*g/mL 5000–2000 mg/kg (toxicity)	Brazil	*Alternaria solani*; *Alternaria alternata*; *Botrytis cinérea*; *Colletotrichum gloeosporioides*; *Mucor hiemalis*; *Phytophthora infestans*; *Venturia pirina*	Crude	[43]
*Caryocar brasiliense* Caryocaracea Pequi	Oil	Agar diffusion test	*In vitro* *In vivo*	Cytotoxicity screening, performed on the *Artemia nauplii*	10 mg/mL	Brazil	*Staphylococcus epidermidis*; *Staphylococcus aureus*; *Pseudomonas aeruginosa*; *Escherichia coli*	Oil	[34]
*Caryocar brasiliense* Caryocaracea Pequi	Leaves	Agar diffusion test/MIC	*In vitro*	—	1.0, 1.5, and 2.0 mg/mL	Brazil	*Enterococcus faecalis*; *Escherichia coli*; *Pseudomonas aeruginosa*; *Staphylococcus aureus*	Crude	[38]
*Caryocar brasiliense* Caryocaracea Pequi	Fruit peels	Agar diffusion test	*In vitro*	—	200–500 mg/mL	Brazil	*Staphylococcus aureus*; *Escherichia coli*	Crude	[44]

^∗^MIC = minimal inhibitory concentration; ^∗∗^MFC = minimal fungicidal concentration.

**Table 3:** Antimicrobial test used in the studies of cashew, caju, and pequi extracts.

Test	References
Minimal inhibitory concentration	[9, 10, 23, 32, 38, 40, 42, 43]
Agar diffusion test	[19, 23, 32, 34, 38, 41, 43, 44]
Antiseptic test	[10]
